# Handpieces for Dental Engines

**Published:** 1882-08

**Authors:** J. B. Hodigkn


*Handpieces* for dental engines are and have been the most vexatious of all appliances connected with modern dentistry. At least such is the experience of the writer. He has run through with most of the handpieces since the first crude "Morrison" came out, though it is certainly true that the newest handpiece is a delightful thing. From the first we have felt that the "chuck" handpiece was the ideal, and ought to be, if properly constructed, superior to any other. Lately Mr. Geo. E. Hodge, of New York, has manufactured a handpiece on the "chuck" style, which after some months use, by self and others seems the nearest approximation to "the thing" we have seen. It is so very simple in its construction it is hardly liable to get out of order, which has been the bane of all the "chuck" handpieces, and its carrying of the bur is certainly steady to perfection. At first it seems a little clumsy but with use this feeling passes away, and the bur is changed as quickly as with any of these devices. The principle on which this handpiece is constructed is certainly the true one, and its merits will tell in the long run.

J. B. Hodigkn.

